# Mycorrhizal specialization for Tulasnellaceae fungi in *Taeniophyllum marianense*, a leafless epiphytic orchid native to Guam

**DOI:** 10.1007/s10265-026-01699-z

**Published:** 2026-03-09

**Authors:** Michael Angelo Paragas Fernandez, Yuki Ogura-Tsujita, Mari Marutani

**Affiliations:** 1 College of Natural and Applied Sciences, University of Guam, Mangilao, Guam 96923 USA; 2 Faculty of Agriculture, Saga University, 1 Honjyo-machi, Saga, 840-8502 Japan; 3 United Graduate School of Agricultural Sciences, Kagoshima University, 1-21-24 Korimoto, Kagoshima, 890-8580 Japan; 4 Present Address: Pacific Biosciences Research Center, University of Hawaiʻi at Mānoa, Honolulu, HI 96822 USA

**Keywords:** Fungal symbiosis, Host tree, Island ecology, Plant establishment, Symbiotic germination, Tulasnellaceae

## Abstract

Orchids are obligately dependent on orchid mycorrhizal fungi (OMF) for nutrition, growth, and establishment. The degree of mycorrhizal specificity varies, from generalists associating with several fungi to specialists relying on a few species. Many leafless epiphytic orchids specifically associate with Ceratobasidiaceae fungi. However, the mycorrhizal associates and specificity of *Taeniophyllum marianense*, a leafless epiphytic orchid native to the remote island of Guam, remain unknown. To address this knowledge gap, we investigated 189 seedling and mature individuals of *T. marianense* growing on 26 host tree species across 10 sites in Guam. OMF were identified using fungal-specific primers targeting Ceratobasidiaceae, Serendipitaceae, Tulasnellaceae, and general Basidiomycota. Sequences were grouped into operational taxonomic units (OTUs) based on 97% similarity. *T. marianense* in Guam associated with a diversity of OMF, including five Tulasnellaceae, six Ceratobasidiaceae, and four Serendipitaceae OTUs. Two Tulasnellaceae OTUs (TU1 and TU2) were the most dominant, comprising 64.6% of fungal sequences and occurring consistently across seedling and mature orchids from different host tree species and habitats, suggesting their role as primary mycorrhizal associates. In contrast, Ceratobasidiaceae and Serendipitaceae were less common and may play minor or opportunistic roles. The mycorrhizal capacity of TU1 and TU2 isolates was confirmed in vitro, where both strains significantly promoted seed germination and protocorm development. While other leafless epiphytic orchids typically associate with Ceratobasidiaceae, *T. marianense* in Guam specifically associates with Tulasnellaceae fungi closely related to globally distributed species. These findings suggest that mycorrhizal specialization may persist in island ecosystems through flexible associations with widespread, locally available fungal associates.

**Supplementary Information:**

The online version contains supplementary material available at 10.1007/s10265-026-01699-z.

## Introduction

Remote oceanic islands provide unique opportunities to investigate the patterns and processes driving symbiosis between plants and other organisms. While a growing body of research on plant-pollinator ecology has provided insights into the evolutionary dynamics of symbiosis during remote island colonization, our understanding of the symbiotic relationships between plants and microorganisms, particularly with mycorrhizal fungi, remains limited (Castro-Urgal and Traveset 2014; Wang et al. 2020). Orchids (family Orchidaceae), one of the most diverse and widespread plant families, offer a compelling system to explore these microbial associations. With over 28,000 species across 763 genera found on oceanic islands and every continent except Antarctica, orchids exhibit remarkable diversity in morphology, growth habit, and life history across terrestrial, epiphytic, and lithophytic environments (Christenhusz and Byng 2016). Notably, epiphytic orchids alone account for 69% of all orchid species (Dearnaley 2007; Zotz 2013).

Despite this morphological and ecological diversity, a common feature among all orchids is their obligate dependence on fungal symbionts known as orchid mycorrhizal fungi (OMF). This dependency on OMF is most evident in orchid seed biology. Though numerous, the dust-like seeds generally lack a nutritive endosperm and therefore rely on colonization by compatible fungi to germinate and establish (Arditti 1992; Rasmussen and Rasmussen 2009; Smith and Read 2008). While the composition of OMF associates may change over time, the symbiosis typically persists into maturity, supplying the orchid host with carbon and other essential nutrients (Bidartondo and Read 2008; Cameron et al. 2006; Fernández et al. 2023; Rasmussen et al. 2015).

Most OMF are basidiomycetous fungi belonging to Tulasnellaceae, Ceratobasidiaceae, and Serendipitaceae which are part of a larger, polyphyletic group *Rhizoctonia*, including pathogenic, saprotrophic, ectomycorrhizal, and endophytic fungi (Oberwinkler et al. 2017; Smith and Read 2008; Zettler and Corey 2018). Tulasnellaceae and Ceratobasidiaceae have been documented as OMF associates across the globe, although their distribution may be uneven at local scales (Jacquemyn et al. 2017; McCormick and Jacquemyn 2013). Due to this variation in distribution, mycorrhizal associations in orchids have been characterized as broader generalists associating with multiple OMF taxa to highly specific involving only a few fungal species (Dearnaley 2007; Jacquemyn et al. 2010; Shefferson 2007; Taylor and Bruns 1997). Both strategies provide competitive advantages wherein generalist associations enable orchids to establish across a broad range of environmental conditions, while specialist associations may reduce competition among co-occurring orchids through niche partitioning (Jacquemyn et al. 2014; Li et al. 2021; Rasmussen et al. 2015; Zotz et al. 2021). While OMF have been well studied around the world, information on their identity and distribution remains limited in remote oceanic islands particularly in the tropical Pacific which boasts some of the highest rates of biodiversity, species endemism, and rare orchid species (Myers et al. 2000). This knowledge gap is especially pronounced within Micronesian islands such as Guam, where orchid-fungal associations have remained largely unexplored (Adair 1971; Cannon et al. 2014; Gilbert et al. 2008; Oberwinkler et al. 2017; Olive 1957; Park et al. 2021).

Guam is the largest and southernmost island of the Mariana Island chain in the Western Pacific Ocean and lies between 13.2° N and 13.7° N and between 144.6° E and 145.0° E with a total area of 549 km^2^, consisting of 34% limestone forest, 14% volcanic, ravine forest, and 18% urban or developed land (Donnegan et al. 2004; Taborosi et al. 2013; Young 1988). To date, 30 orchid species have been documented in the Mariana Islands with the majority being epiphytic (Raulerson and Rinehart 1992; Stone 1970). The leafless epiphytic orchid *Taeniophyllum marianense* Schltr. is one of the most ubiquitous and has been observed in a wide range of habitats in Guam and Micronesia (Fosberg and Sachet 1987; Raulerson and Rinehart 1992; Stone 1970). Like other leafless epiphytic orchids, the roots of *T. marianense* contain cortical cells with chloroplasts and stomatal complexes, allowing them to perform photosynthesis in the absence of leaves (Carlsward et al. 2006). Although the evolution towards leaflessness in orchids is often attributed to xeric adaptation, obligate mycorrhizal associations may also be linked to this leafless lifestyle (Qin et al. 2021; Rammitsu et al. 2019). In previous studies, Ceratobasidiaceae fungi have been identified as exclusive mycorrhizal associates in related leafless orchid species such as *Taeniophyllum glandulosum* and *T. obtusum* from China, Japan, and Indonesia (Irawati et al. 2009; Qin et al. 2021; Rammitsu et al. 2019; Yagame and Yamato 2009). Similar patterns of fungal specificity to Ceratobasidiaceae have been observed in other leafless orchid genera including *Chiloschista*, *Phalaenopsis*, *Dendrophylax*, and *Campylocentrum* across Asia and the Americas (Hoang et al. 2017; Johnson et al. 2023; Mújica et al. 2018; Otero et al. 2002; Qin et al. 2021). However, the mycorrhizal associations of *T. marianense*, a leafless epiphyte native to Guam, remain largely unknown, and it is unclear whether this species exhibits the same degree of fungal specificity observed in related orchid species.

In this study, we investigated the host tree and mycorrhizal associations in *T. marianense* in Guam to assess the hypothesis that *T. marianense* is a mycorrhizal generalist associating with a broad range of host trees and OMF in an island system. We used molecular and phylogenetic approaches to characterize these associations and conducted in vitro symbiotic germination experiments to assess the mycorrhizal capacity of OMF isolated from *T. marianense* roots. Our findings offer insight into the role of symbiotic relationships in shaping orchid distribution and survival on remote oceanic islands, as well as the persistence of these mutualisms during island colonization. Such knowledge carries important implications for the conservation of unique and often rare island endemic species.

## Materials and methods

### Study sites

**Fig. 1:**
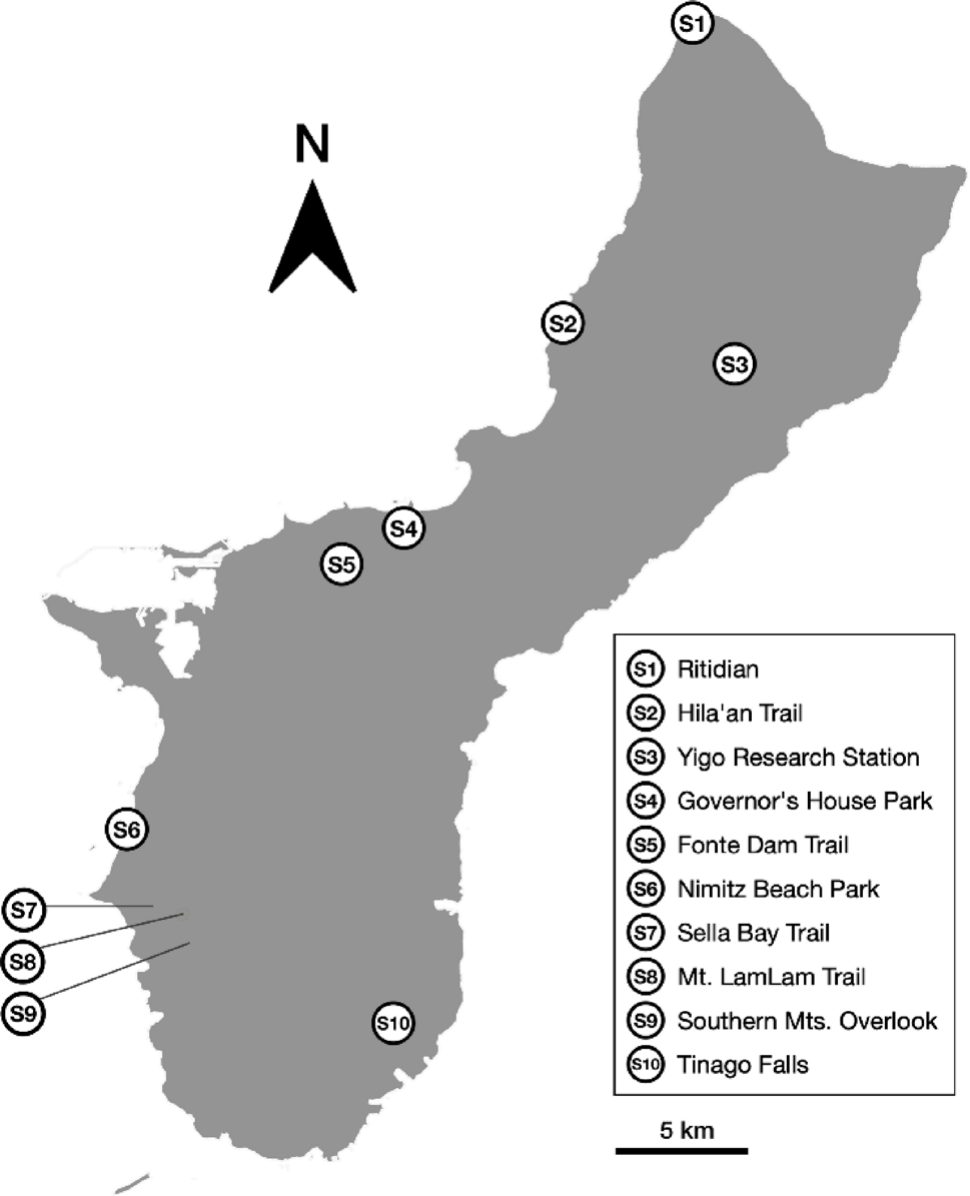
Map of *T. marianense* sampling sites in Guam. A detailed description of sampling sites is provided in Table 1

Ten sites in Guam were surveyed from August 2021 to February 2022, covering the predominantly limestone forest habitat of the North, volcanic ravine forest of the South, and planted trees in developed areas illustrated in Fig. 1. Tables 1 and 2 shows geographic coordinates, habitat and host tree species identified at each study site.

**Table 1:** Description of study sites, including geographic coordinates, habitat type, the number of *T. Marianense* individuals sampled, and the number of root samples with mycorrhizal colonization collected from August 2022 and February 2023. Locations of all study sites are also illustrated in Fig. 1

Site no.	Locality	Geographic coordinates		Habitat	No. of orchid individuals sampled	No. of root samples with mycorrhizal colonization	
		°*N*	°E			From mature plants	From seedlings
S1	Ritidian	13.65	144.86	Limestone Forest	8	21	2
S2	Hila’an Trail	13.54	144.81	Limestone Forest	12	34	3
S3	Yigo Research Station	13.53	144.87	Limestone Forest	44	50	35
S4	Governor’s House Park	13.47	144.75	Developed Land	20	23	15
S5	Fonte Dam Trail	13.46	144.73	Volcanic/Ravine Forest	36	50	25
S6	Nimitz Beach Park	13.36	144.65	Developed Land	11	7	8
S7	Sella Bay Trail	13.34	144.66	Developed Land	18	26	9
S8	Mt. LamLam Trail	13.33	144.67	Volcanic/Ravine Forest	12	14	8
S9	Southern Mts. Overlook	13.32	144.67	Developed Land	25	22	20
S10	Tinago Falls	13.30	144.75	Volcanic/ Ravine Forest	3	8	0
				Total	189	255	125

**Table 2:** Number of *T. Marianense* individuals collected from different host trees across 10 sites

Clade	Family	Host tree species	No. of *T. marianense* individuals sampled at each site										
			S1	S2	S3	S4	S5	S6	S7	S8	S9	S10	Total
Eudicots	Fabaceae	*Leucaena leucocephala* ^a^			4		5			1			10
		*Delonix regia* ^a^				1		6^b^		3			10
		*Acacia confusa* ^a^							3	4			7
		*Pterocarpus indicus* ^a^				3			2		1		6
		*Cynometra ramiflora*			3								3
		*Tamarindus indica* ^a^				3							3
	Lamiaceae	*Vitex parviflora* ^a^			11		7	1					19
		*Premna serratifolia*			7								7
	Calophyllaceae	*Calophyllum inophyllum*		1				1			15	3	20
	Rubiaceae	*Morinda citrifolia*			11		5						16
	Anacardiaceae	*Mangifera indica*				7	6						13
	Malvaceae	*Hibiscus tiliaceus*			5		3			1			9
	Moraceae	*Artocarpus altilis*		5		4							9
	Lythraceae	*Lagerstroema indica* ^a^									7		7
	Casuarinaceae	*Casuarina equisetifolia*		3						2			5
	Sapotaceae	*Manilkara zapota* ^a^				1			3				4
	Oxalidaceae	*Averrhoa bilimbi*			1		2						3
	Phyllanthaceae	*Phyllanthus mariannensis*			2					1			3
	Boraginaceae	*Cordia subcordata*		2									2
	Apocynaceae	*Cascabela thevetia* ^a^							1				1
	Bignoniaceae	*Spathodea campanulata* ^a^							1				1
	Lecythidaceae	*Barringtonia asiatica*		1									1
Monocots	Arecaceae	*Cocos nucifera*	8				1	3^c^	8		1		21
		*Heterospathe elata* ^a^					5						5
		*Adonidia merrillii* ^a^									1		1
	Pandanaceae	*Pandanus tectorius*					2						2
On a fallen branch of unidentified species						1							1
		Total	8	12	44	20	36	11	18	12	25	3	189

^a^Introduced species to Guam (Stone 1970; Yoshioka 2008 )

^b^Source of isolated TU1 strain

^c^Source of isolated TU2 strain

### Sampling of orchid roots 

**Fig. 2:**
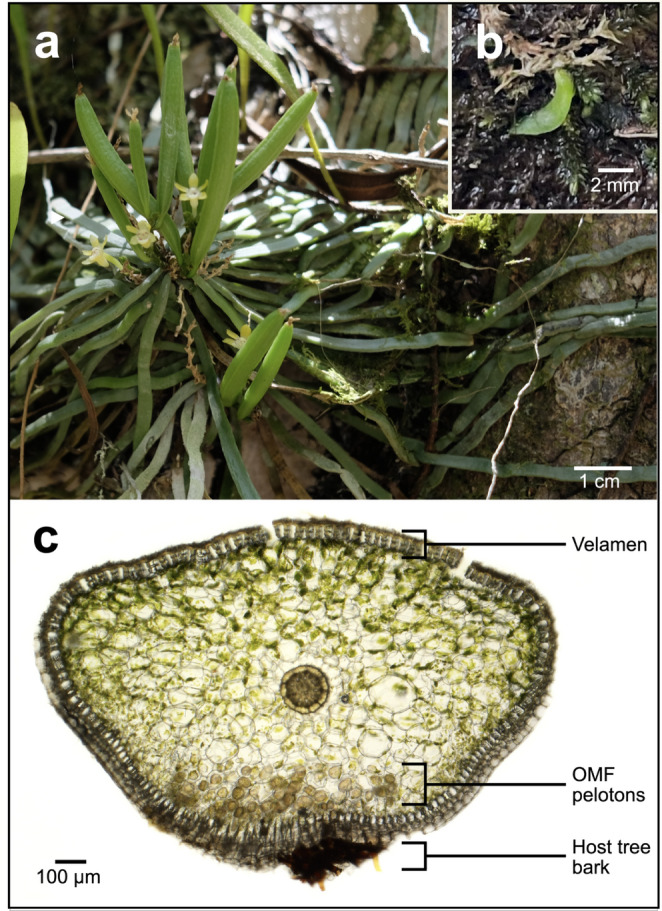
*T. marianense. ***a** Mature individual with flowers and fruits observed in situ. **b** Spontaneous seedling observed in situ. **c** Transverse section of root showing orchid mycorrhizal fungi (OMF) pelotons colonizing root cortex cells near the point of root that attaches to the host tree bark

Similar to other *Taeniophyllum* species, the leafless *T. marianense* undergoes a distinct development following germination wherein protocorms appear ribbon-like and continue to elongate, allowing for easy identification of spontaneous seedlings in situ on host tree bark (Rammitsu et al. 2019; Renner and Beadel 2011). Fig. 2 shows a well-established adult form (Fig. 2a) and protocorm (Fig. 2b). Maturity was classified into two groups based on the stage of plant development: (1) mature, well-established plants with signs of reproduction (presence or indication of flower or fruit production), and (2) seedlings with at least three developed roots present without signs of reproduction. In this study, a total of 99 mature plants and 90 spontaneous seedlings of *T. marianense* were examined from the 10 sites spanning 43 km across Guam. Three to four root samples were collected from each mature plant and entire seedlings were collected. We examined roots in direct contact to the host tree bark as mycorrhizal colonization is more likely to occur (Suárez et al. 2006).

The root samples were washed under running tap water and further scrubbed under sterile distilled water using a Leica EZ4 stereomicroscope (Leica, Wetzlar, Germany) to remove any bark or plant debris. Each root was hand-sectioned along its entire length and inspected under a Nikon AZ100 stereomicroscope (Nikon, Tokyo, Japan) to confirm mycorrhizal colonization by the presence of OMF pelotons within cortex cells near the point of attachment to the bark (Fig. 2c). Up to ten root cross-sections containing OMF pelotons were collected per sample, with the outer velamen carefully removed using a sterile scalpel. Root cross-sections were rinsed 2–3 times with sterile Tris-EDTA (TE) buffer and stored in fresh TE buffer at -20 °C until further processing. A total of 380 mycorrhizal samples were collected from 255 roots from mature plants and 125 roots from seedlings (Table 1).

### Molecular identification of mycorrhizal fungi

OMF pelotons were released from root cortical cells by macerating root cross-sections in a sterile Kimble Biomasher II Closed System Micro Tissue Homogenizer (Fisher Scientific, Hampton, NH, USA). Total genomic DNA was extracted from the homogenate using the Sigma-Aldrich Extract-N-Amp Plant PCR Kit (Sigma-Aldrich, St. Louis, MO, USA) according to the manufacturer’s instructions. The internal transcribed spacer (ITS) region of the nuclear ribosomal DNA was amplified in samples using the Basidiomycota-specific primer pair ITS1F/ITS4B (Gardes and Bruns 1993). Because this primer pair has limited coverage within major OMF groups, we also used Tulasnellaceae-specific primers ITS5/ITS4-Tul2 (Oja et al. 2015; White et al. 1990), Ceratobasidiaceae-specific primers CeTh1/CeTh4 (Porras-Alfaro and Bayman 2007), and Serendipitaceae-specific primers ITS3Seb/TW13 (Selosse et al. 2007) to cover most of the major families of OMF. While ITS5/ITS4-Tul2 can amplify the whole ITS region, primer pairs CeTh1/CeTh4 and ITS3Seb/TW13 only partially amplify ITS1 and 5.8 S, and ITS2 and 28 S regions, respectively. PCR amplification was done in a total reaction volume of 16 µL consisting of 2 µL of sample DNA, 8.5 µL of 1x Qiagen Multiplex PCR master mix (Qiagen, Germany), 4.5 µL of sterile water, and 0.5 µL of each primer (5 µM). The PCR protocol consisted of an initial denaturation at 94 °C for 3 min; 35 cycles of denaturation at 94 °C for 30 s, annealing at 57 °C for 90 s, and extension at 72 °C for 90 s; and a final extension at 72 °C for 10 min. The PCR was carried out in a Bio-Rad T100 thermal cycler (Bio-Rad, Hercules, CA, USA). The PCR products were then analyzed by electrophoresis on 1% agarose gel stained with GelGreen (Biotium, Hayward, CA, USA) and viewed under a UV light to verify proper amplicon size and purity. PCR products with multiple amplicons were individually excised and purified from agarose according to Abraham et al. (2017). The PCR products were purified and sequenced by Genewiz/Azenta (Azenta, South Plainfield, NJ, USA). All sequences were checked and manually adjusted using Geneious Prime version 2024.0.4 (website https://www.geneious.com*).* Identical sequences from a single sample detected using different primer pairs were discarded. Sequences were then subjected to a BLAST search for fungal taxonomic identification against the NCBI (National Center for Biotechnology Information) GenBank database. Sequences assignable to major OMF groups Tulasnellaceae, Ceratobasidiaceae, and Serendipitaceae were binned into operational taxonomic units (OTUs) based on 97% sequence similarity. Representative sequences of each OTU were deposited in the NCBI GenBank database for Tulasnellaceae (PP938874–PP938878), Ceratobasidiaceae (PP938889–PP938894) and Serendipitaceae (PP916010–PP916013).

### Phylogenetic analyses

Separate phylogenetic analyses were conducted on the identified Tulasnellaceae, Ceratobasidiaceae, and Serendipitaceae OTUs. BLASTn hits of OMF ITS sequences showing > 95% sequence similarity and > 95% query coverage to each OTU were included in each analysis. To investigate the phylogenetic placement of the OTUs to known fungal species, reference sequences were included to cover Tulasnellaceae (Arifin et al. 2021, 2022; Calevo et al. 2020; Freitas et al. 2020; Rammitsu et al. 2021a), Ceratobasidiaceae (Cruz et al. 2022; Pujasatria et al. 2022), and Serendipitaceae (Fritsche et al. 2021; Zhang et al. 2022). Reference sequences of OMF from other *Taeniophyllum* species and other leafless epiphytic orchids were also included (Otero et al. 2002; Rammitsu et al. 2019; Unruh et al. 2019; Yagame and Yamato 2009). Sequences of *T. helicospora* were selected as an outgroup for Tulasnellaceae, sequences of *Botryobasidium* were selected as an outgroup for Ceratobasidiaceae, and sequences of *Tremiscus helvelloides* and *Auricularia auricula-judae* were selected as an outgroup for Serendipitaceae. The sequences for each analysis were aligned using MAFFT (Katoh and Standley 2013). Each phylogenetic tree was constructed using the maximum likelihood method based on the GTR + G + I model on RAxML-NG (Stamatakis 2014). The relative robustness of each branch was estimated using the bootstrap method with 1,000 replicates (Felsenstein 1985).

### In vitro symbiotic culture

Fungal isolates were recollected from the two most common OTUs identified in *T. marianense*, Tulasnellaceae OTUs TU1 and TU2, from the roots of two mature plants at Site S6. OMF pelotons were extracted from mycorrhizal roots following Rammitsu et al. (2019) and cultured in sterile Petri dishes containing water agar medium supplemented with 50 ppm of chloramphenicol. Fungal cultures were incubated in the dark at ambient temperature (25 ± 3 °C) for 1–5 days. Hyphal tips growing from pelotons were excised using a sterile scalpel and sub-cultured onto Petri dishes containing potato dextrose agar (PDA; Difco, Sparks, MD, USA). This process was repeated until successful isolation of a single fungal strain that was free of other microbial contamination. The identity of fungal isolates to TU1 and TU2 was reconfirmed using previously described molecular methods. Pure isolates were obtained within 4 weeks for the in vitro symbiotic culture experiments.

*T. marianense* seeds from Site S9 were used for the in vitro symbiotic culture experiments. Mature green seed capsules were collected prior to natural dehiscence and surface-sterilized in 5% sodium hypochlorite with Tween for 5 min, rinsed twice with sterile distilled water, dipped in 95% ethanol for 5 s, and allowed to air-dry on a sterile Petri dish. The seed capsules were then excised longitudinally and seeds were collected into a microcentrifuge tube containing 1 mL of sterile distilled water. The seed-water solution was then pipetted onto a Petri dish containing water agar medium in 1 cm sub-sections containing 30–50 seeds. The seeds were incubated for 1 week and screened for contamination under a Nikon AZ100 stereomicroscope. Each aseptic sub-section containing clean seeds was cut and transferred onto a new Petri dish containing oatmeal agar (OA; Rammitsu et al. 2023) with an average of 42 seeds for each dish. Seed viability was inspected under a stereomicroscope by checking for the presence of a visible embryo; only seeds containing a visible embryo were assessed in culture, while seeds lacking embryos were excluded from assessment.

**Fig. 3:**
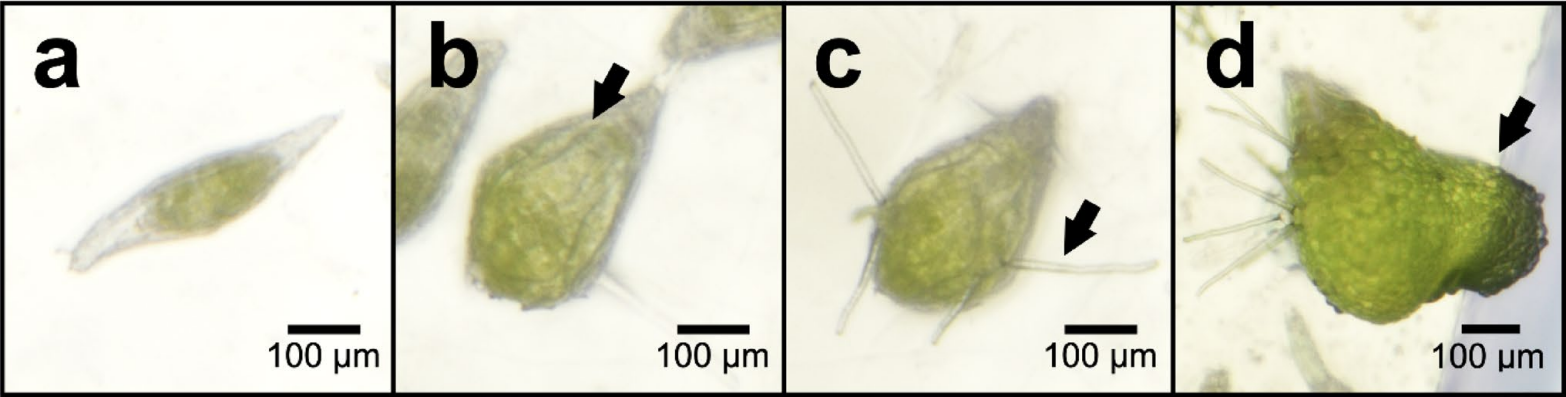
Stages of development of *T. marianense* seeds germinated in vitro. **a** Stage 1, no germination. **b** Stage 2, enlargement of embryo with ruptured seed coat (arrow). **c** Stage 3, development of rhizoids (arrow). **d** Stage 4, enlargement of protocorm (arrow)

A one cm plug of PDA medium containing actively growing hyphal tips of precultured fungal isolates TU1 and TU2 was excised and inoculated onto the OA medium. The fungal plug was spaced about 25 mm from the seed plug. Cultures without fungal inoculations served as the control. There were seven replicates for each treatment. The in vitro symbiotic germination experiment was conducted under a 12 h light / 12 h dark photoperiod at 25 ± 3 °C. After 33 days, the total number of seeds at four different stages of seed germination and development (Fig. 3) was counted, where Stage 1 = no germination (Fig. 3a), Stage 2 = enlarged seed embryo with ruptured seed coat (Fig. 3b), Stage 3 = rhizoid formation (Fig. 3c), and Stage 4 = elongation of protocorm (Fig. 3d). The percentage of seeds at each stage was calculated by dividing the number of seeds observed at a given stage by the total number of seeds tested, then multiplying the result by 100.

### Statistical analyses

Differences in putative mycorrhizal community composition between *T. marianense* collected from different host tree species were visualized with nonmetric multidimensional scaling (NMDS) using Bray–Curtis dissimilarity matrices. For the in vitro symbiotic culture experiments, the percentage of successfully germinated seeds was calculated:

$$\begin {aligned} &\mathrm{T}\mathrm{h}\mathrm{e}\:\mathrm{p}\mathrm{e}\mathrm{r}\mathrm{c}\mathrm{e}\mathrm{n}\mathrm{t}\mathrm{a}\mathrm{g}\mathrm{e}\:\left(\mathrm{\%}\right)\mathrm{o}\mathrm{f}\:\mathrm{s}\mathrm{u}\mathrm{c}\mathrm{c}\mathrm{e}\mathrm{s}\mathrm{s}\mathrm{f}\mathrm{u}\mathrm{l}\mathrm{l}\mathrm{y}\:\mathrm{g}\mathrm{e}\mathrm{r}\mathrm{m}\mathrm{i}\mathrm{n}\mathrm{a}\mathrm{t}\mathrm{e}\mathrm{d}\:\mathrm{s}\mathrm{e}\mathrm{e}\mathrm{d}\mathrm{s} \\ =&\:\frac{[\mathrm{S}\mathrm{t}\mathrm{a}\mathrm{g}\mathrm{e}\:2\:+\:\mathrm{S}\mathrm{t}\mathrm{a}\mathrm{g}\mathrm{e}\:3\:+\:\mathrm{S}\mathrm{t}\mathrm{a}\mathrm{g}\mathrm{e}\:4]}{\left[\mathrm{t}\mathrm{o}\mathrm{t}\mathrm{a}\mathrm{l}\:\mathrm{n}\mathrm{u}\mathrm{m}\mathrm{b}\mathrm{e}\mathrm{r}\:\mathrm{o}\mathrm{f}\:\mathrm{s}\mathrm{e}\mathrm{e}\mathrm{d}\mathrm{s}\right]}\:\times\:\:100 \end {aligned} $$

A one-way ANOVA was performed to compare the effect of mycorrhizal inoculation on the percentage of seeds germinated. Statistical analyses and data visualization were done in R (ver. 3.4.1) with the packages ‘vegan’ (Oksanen et al. 2022) and ‘ggplot2’ (Wickham 2016).

## Results

We observed 189 *T. marianense* orchids growing on 91 individual host trees of 26 different species during the study (Tables 1 and 2). Of the host trees observed, 12 are introduced species to Guam (Stone 1970; Yoshioka 2008). *T. marianense* was found marginally higher on *Cocos nucifera* (Arecaceae) at 10.5%, *Calophyllum inophyllum* (Calophyllaceae) at 9.5% and the invasive tree species *Vitex parviflora* (Lamiaceae) at 11.6% of all trees surveyed. The other 23 host tree species included *Acacia confusa*, *Artocarpus altilis*, *Adonidia merrillii*, *Averrhoa bilimbi*,* Barringtonia asiatica*, *Cascabela thevetia*, *Casuarina equisetifolia*, *Cordia subcordata*, *Cynometra ramiflora*, *Delonix regia*, *Heterospathe elata*, *Hibiscus tiliaceus*, *Lagerstroema indica*, *Leucaena leucocephala*, *Mangifera indica*, *Manilkara zapota*, *Morinda citrifolia*, *Pandanus tectorius*, *Pterocarpus indicus*, *Premna serratifolia*, *Phyllanthus mariannensis*, *Spathodea campanulata*, and *Tamarindus indica* (Table 2).

**Fig. 4:**
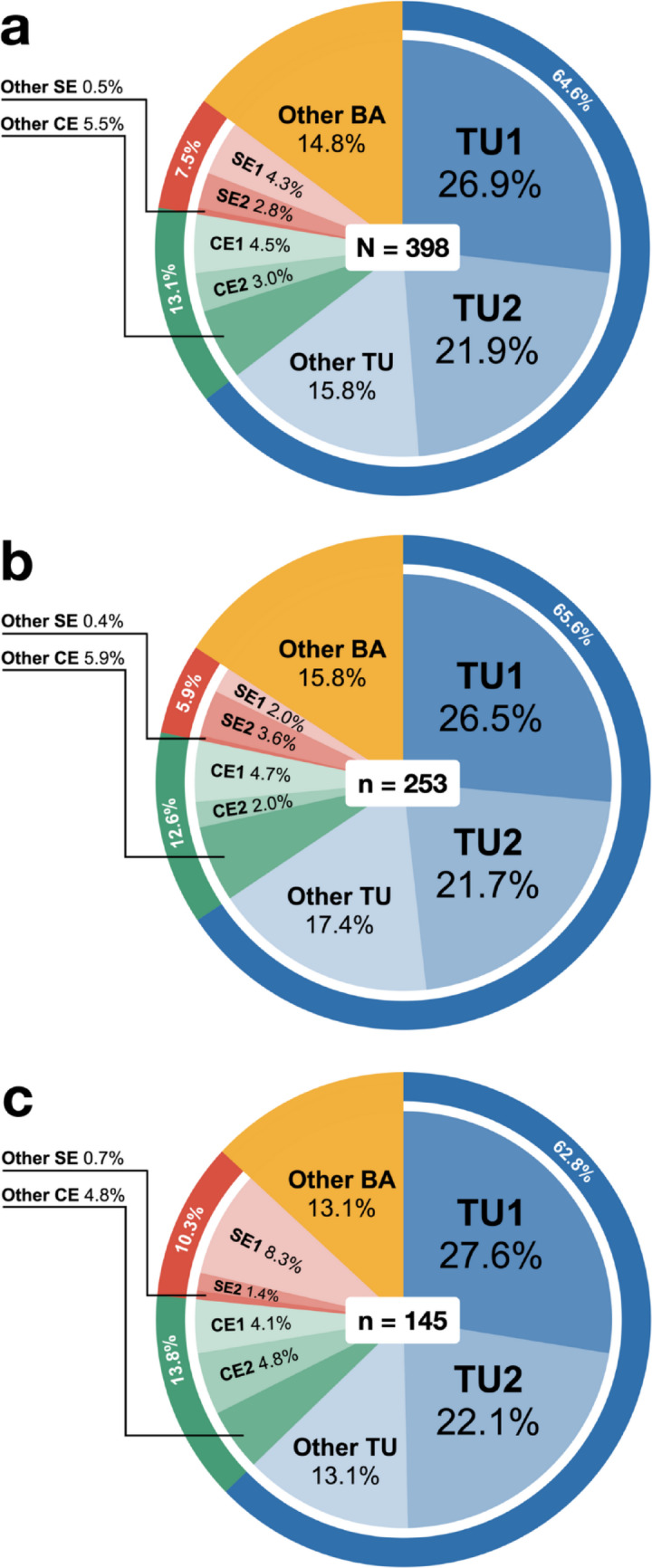
Frequency distribution of operational taxonomic units (OTUs) assigned to Tulasnellaceae (TU), Ceratobasidiaceae (CE), Serendipitaceae (SE), and other Basidiomycota (BA) detected from *T. marianense* root samples. Fungal ITS amplicon sequences from root samples were clustered into OTUs based on 97% sequence similarity. OTU proportions are shown for root samples from **a** all stages (*n* = 398 sequences), **b** mature plants (*n* = 253), and **c** seedlings (*n* = 145)

A total of 398 fungal DNA sequences were obtained from 380 mycorrhizal root samples of mature and seedling *T. marianense.* In 91 root samples, two to four different fungi were detected using different primer sets. Fungal DNA sequences were identified to Tulasnellaceae (64.6%), Ceratobasidiaceae (13.1%), and Serendipitaceae (7.5%), and the remaining 14.8% were other Basidiomycota (Table S1). Tulasnellaceae sequences consisted of five OTUs (TU1–TU5), wherein the two most dominant TU1 and TU2 accounted for 48.8% of all fungal sequences combined, with each comprising 26.9% and 21.9% respectively (Fig. 4a). This pattern of predominant mycorrhizal association with TU1 and/or TU2 was apparent in both mature (Fig. 4b) and seedling (Fig. 4c) *T. marianense* at most sampling sites. The other Tulasnellaceae OTUs included TU3 (7.8%), TU4 (7.3%), and TU5 (0.7%). Ceratobasidiaceae sequences consisted of six OTUs (CE1–CE6), and the most frequent Ceratobasidiaceae OTUs, CE1 and CE2 accounted for 4.5% and 3.0% of sequences, respectively (Fig. 4a). Serendipitaceae sequences consisted of four OTUs (SE1–SE4) and the most frequent Serendipitaceae OTU SE1 was present in 4.3% of sequences (Fig. 4a). Other Basidiomycota sequences consisted of 14.8% sequences.

**Fig. 5:**
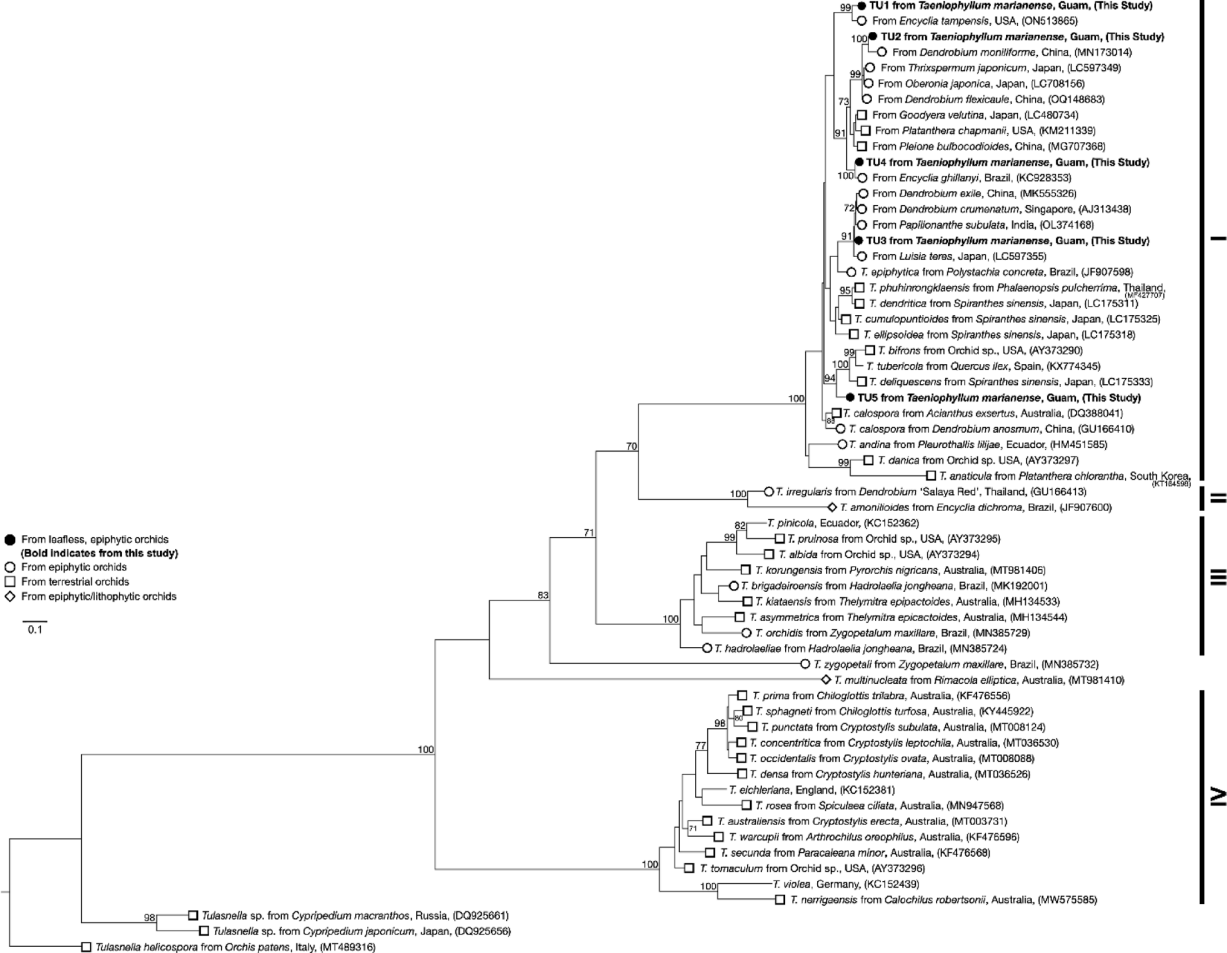
Maximum likelihood tree for Tulasnellaceae OTUs identified in *T. marianense* using complete ITS and 5.8 S regions. Symbols indicate type of orchid host. No symbol indicates an unknown or non-orchid origin. Only bootstrap values greater than 70% are shown. *Tulasnella helicospora* was selected as the outgroup taxa. The tree is drawn to scale and branch length is proportional to the number of substitutions per site. The final dataset comprised 831 bp. Four major Tulasnellaceae groups (I-IV) were identified with high bootstrap support (100% BS) with all identified OTUs in our study grouped into Group I

Phylogenetic analysis of all Tulasnellaceae OTUs including ITS sequences of the closest BLASTn hits and reference *Tulasnella* species consisted of 61 sequences with a final dataset of 831 bp (Fig. 5). The Tulasnellaceae maximum likelihood tree showed four well supported groups (100% BS). All Tulasnellaceae OTUs identified in our study grouped into Group I, nested within *Tulasnella anaticula*,* T. danica*, and *T. andina* with a 100% BS. The most dominant TU1 formed a well-supported, monophyletic clade with mycorrhizal fungi identified from *Encyclia tampensis*, a widespread epiphytic orchid native to Florida and the Bahamas (99% BS) (Garcia et al. 2024). TU2 and TU4 formed a monophyletic clade with OMF identified in epiphytic and terrestrial species (91% BS). TU2 was 99% identical to mycorrhizal fungi identified in *Dendrobium moniliforme* from China (Meng et al. 2019), *Thrixspermum japonicum* from Japan (Rammitsu et al. 2021b), *Oberonia japonica* from Japan (Rammitsu et al. 2023), and *Dendrobium flexicaule* from China (Wang and Gao, unpublished) (99% BS). TU4 was 99% identical to OMF from the epiphytic *Encyclia ghillanyi* from Brazil (KC928353). TU3 formed a monophyletic clade mainly consisting of OMF from epiphytic orchids *Dendrobium exile* from China (MK555326), *Dendrobium crumenatum* from Singapore (AJ313438), *Papilionanthe subulata* from India (​​OL374168), and *Luisia teres* from Japan (LC597355) (91% BS). TU5 formed a monophyletic clade with *Tulasnella bifrons* from a terrestrial orchid from the United States (AY373290), *T. tubericola* from *Quercus ilex* from Spain (KX774345), and *T. deliquescens* from *Spiranthes sinensis* from Japan (LC175333) (94% BS).

**Fig. 6:**
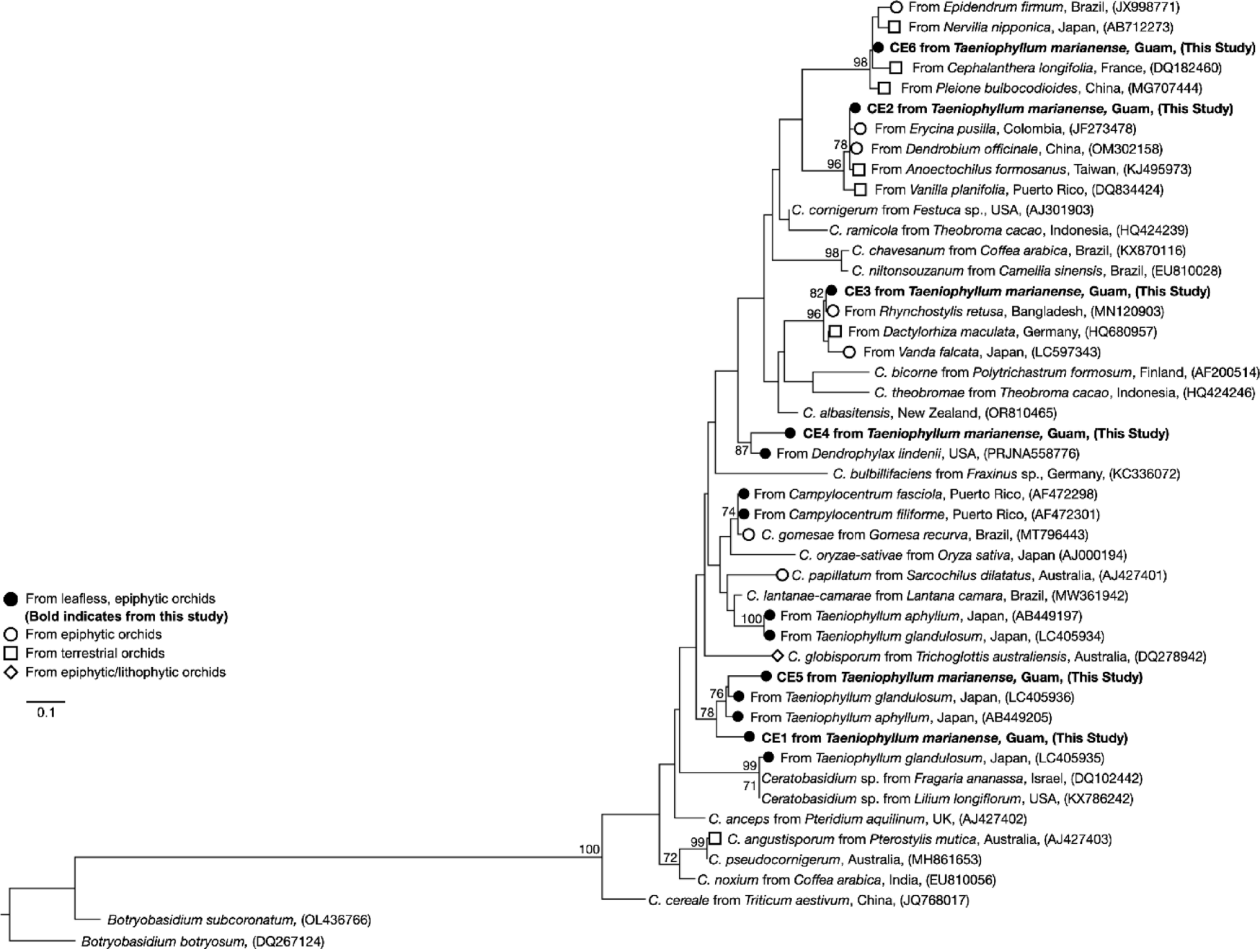
Maximum likelihood tree for Ceratobasidiaceae OTUs identified in *T. marianense* using partial ITS1 and complete 5.8 S regions. Symbols indicate type of orchid host. No symbol indicates an unknown or non-orchid origin. Only bootstrap values greater than 70% are shown. *Botryobasidium botryosum* and *B. subcoronatum* were selected as the outgroup taxa. The tree is drawn to scale and branch length is proportional to the number of substitutions per site. The final dataset comprised 237 bp

Phylogenetic analysis of Ceratobasidiaceae yielded a maximum likelihood tree of 47 sequences with a final dataset of 237 bp (Fig. 6). The most dominant Ceratobasidiaceae OTU CE1 formed a monophyletic clade with CE5 which also included mycobionts identified from *T. glandulosum* (Rammitsu et al. 2019; Yagame and Yamato 2009) from Japan (78% BS). CE2 formed a well-supported monophyletic clade with *Erycina pusilla* from Colombia (JF273478), *Dendrobium officinale* from China (OM302158), *Anoectochilus formosanus* from Taiwan (KJ495973), and *Vanilla planifolia* from Puerto Rico (DQ834424) (96% BS). Though not as common, CE4 was 87% identical to *Ceratobasidium* isolated from the critically endangered, leafless epiphytic orchid *Dendrophylax lindenii* native to Florida and the Bahamas (Unruh et al. 2019). Phylogenetic analysis of the least detected OMF family Serendipitaceae produced a maximum likelihood tree constructed with 53 sequences and a final dataset of 537 bp (Fig. S1). All Serendipitaceae OTUs grouped within the *Serendipita* clade sister to *Sebacina*. The most frequent OTU SE1 shared 98% sequence identity with OMF isolated from an epiphytic *Stelis* sp. from Ecuador (HM451823) and the terrestrial *Neottia cordata* from France (KJ188508).

**Fig. 7:**
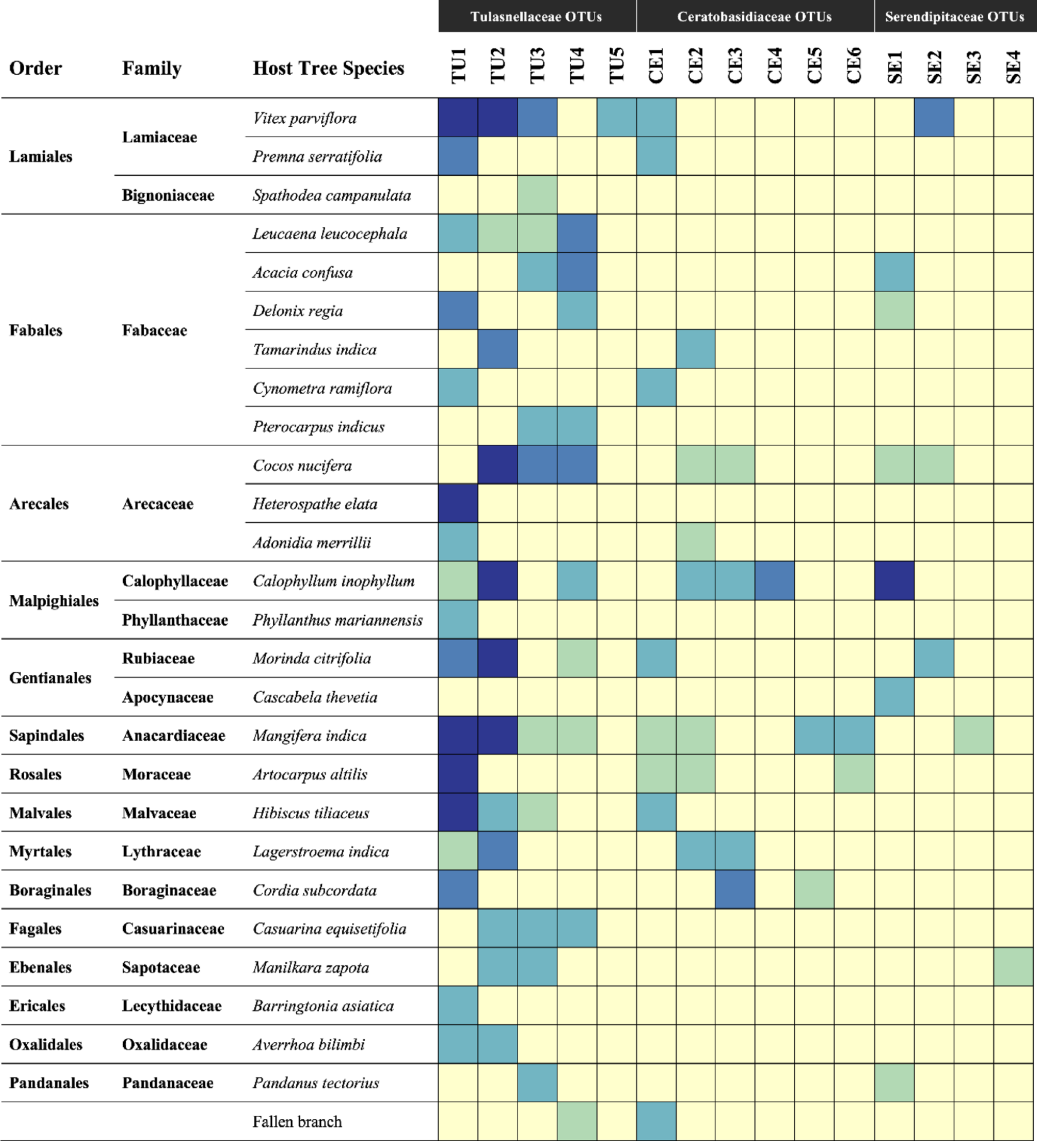
Heatmap of detected mycorrhizal OTUs (columns) between different host tree species (rows) identified in *T. marianense.* The magnitude of detected mycorrhizal OTUs is represented by a yellow to blue color gradient

Mycorrhizal incidence of OMF OTUs by the different host tree species is illustrated as a heatmap in Fig. 7. OMF OTUs were identified in 85.7% of all host trees sampled (78/91). Comparison of mycorrhizal fungi among different host trees showed that TU1 was the most frequently detected, present in 43.6% (34/78) of host trees with OMF. TU2 closely followed at about 35.9% (28/78) of OMF host trees. Together, the two Tulasnellaceae OTUs accounted for most host trees sampled at 79.5% (62/78). When comparing OMF OTUs by the different host tree species, TU1 was exclusively detected in nine host tree species: *Adonidia merrillii*,* Artocarpus altilis*,* Barringtonia asiatica*,* Cordia subcordata*,* Cynometra ramiflora*,* Delonix regia*,* Heterospathe elata*,* Phyllanthus mariannensis*, and *Premna serratifolia.* TU2 was exclusively detected in four host tree species: *Casuarina equisetifolia*,* Cocos nucifera*,* Manilkara zapota*, and *Tamarindus indica*. Both TU1 and TU2 were detected in eight host tree species: *Averrhoa bilimbi*,* Hibiscus tiliaceus*,* Lagerstroema indica*,* Leucaena leucocephala*,* Mangifera indica*,* Morinda citrifolia*, including the most commonly identified *Calophyllum inophyllum* and *Vitex parviflora.* The remaining OTUs showed no consistent pattern and were randomly detected in different host tree species. The NMDS ordination also did not show any distinguishable pattern in OMF community composition by the different host tree species (Fig. S2).

**Fig. 8:**
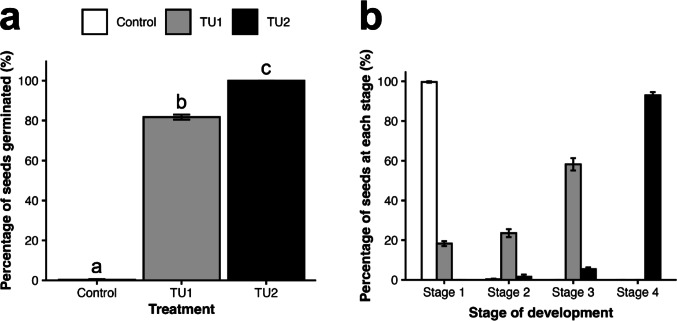
Effects of Tulasnellaceae isolates TU1 and TU2 on seed germination and protocorm development of *T. marianense* after 33 days of symbiotic culture, including without inoculants as a control. **a** Percentage of seeds at each stage of development wherein Stage 1 = no germination, Stage 2 = enlarged seed embryo, Stage 3 = rhizoid formation, and Stage 4 = elongation of protocorm. **b** Comparison of percentage of seeds germinated ([Stage 2 + Stage 3 + Stage 4]/[total number of seeds]) between inoculation treatments TU1 and TU2 and without inoculation as a control. Bars represent standard error of mean (SEM) and different letters indicate significantly different values (*p* < 0.001). There was a total of seven replicates per treatment with an average of 42 (30–50) seeds per replicate

For the in vitro symbiotic germination experiments, we successfully isolated two of the most dominant Tulasnellaceae OTUs of *T. marianense*: TU1 from an individual growing on *Delonix regia* and TU2 from an individual growing on *Cocos nucifera*, both collected from roots of mature plants at Site S6 (Table 2). OTU assignment was confirmed by molecular methods. Seeds collected from Site S9 were used for germination trials, with seed viability estimated at approximately 60%. Germination percentages reported are based only on seeds containing visible embryos. In vitro symbiotic germination tests with the two fungal isolates confirmed their mycorrhizal capacity (Fig. 8). After 33 days of symbiotic culture, TU2 promoted the greatest seed germination and protocorm development, with more than 93% of viable seeds reaching Stage 4 of advanced protocorm development (Fig. 3d). TU1 promoted seed germination to a lesser extent with 58% of seeds reaching Stage 3 (Fig. 3c), 24% of seeds reaching Stage 2 (Fig. 3b), and 18% of seeds remaining ungerminated at Stage 1 (Fig. 3a). In the control treatment without any fungal inoculants, more than 99% of viable seeds remained ungerminated. A one-way ANOVA revealed that there was a statistically significant difference in the proportion of seeds germinated between treatments (*F* (2, 18) = 5106, *p* < 0.001) (Fig. 8b). A Tukey’s Honestly Significant Difference (HSD) test revealed significant differences among all treatment groups and post-hoc comparisons indicated that all treatments were significantly different from each other (*p* < 0.001).

## Discussion

This study presents the first report on the host tree preferences, mycorrhizal associations, and symbiotic capacity in the leafless epiphytic orchid, *T. marianense*, native to Guam. We found 189 *T. marianense* growing on 26 different host tree species belonging to 18 families (Table 2). The host species not only included native trees such as *Cocos nucifera* and *Calophyllum inophyllum* but also introduced plants including the invasive *Vitex parviflora* — a relatively recent introduction that has become increasingly naturalized across the island. Many of the identified host species have been documented among the most abundant trees distributed in Guam based on previous plant surveys (Donnegan et al. 2004; Lazaro et al. 2020). These findings suggest that *T. marianense* exhibits a generalist strategy in host tree selection, establishing itself on a broad range of tree species. This ecological generalism likely contributes to its relatively widespread distribution across Guam, where it is found in various ecosystems from native limestone and volcanic ravine forests to disturbed and urban areas (Table 1). Previous reports have similarly noted the presence of this orchid across both natural forests and disturbed habitats in Guam (Raulerson and Rinehart 1992; Stone 1970; Taborosi et al. 2013).

Despite this broad host range, *T. marianense* may still be selectively associating with particular host tree traits that enhance facilitation. The success of orchid germination and establishment is influenced by a range of abiotic factors including light availability, moisture, substrate chemistry, and substrate texture (Rasmussen et al. 2015). As a leafless, and observed sunlight-tolerant epiphyte, *T. marianense* may be undergoing range expansion driven by preference for specific host tree traits that promote increased light exposure such as canopy openness, leaf phenology, or disturbed habitats. In particular, large trees with rough bark and high water-retention capacity, features observed in many host trees in this study, are known to enhance seed capture and promote germination and establishment of dust-like orchid seeds (Adhikari and Fischer 2012; Callaway et al. 2002; Zarate-García et al. 2020). Beyond these structural advantages, host trees may also be indirectly shaping orchid distribution by creating microenvironments that facilitate the persistence of key mycorrhizal fungi essential for orchid development (Otero et al. 2007a; Rasmussen 2002).

In characterizing the OMF associations of *T*. *marianense* in Guam, we identified a total of 398 fungal sequences from mature and protocorm root samples representing a diversity of Tulasnellaceae, Ceratobasidiaceae, Serendipitaceae, and other Basidiomycota. The majority of sequences were identified to Tulasnellaceae at 64.6% of all sequences with two dominant operational taxonomic units (OTUs), TU1 and TU2, accounting for 26.9% and 21.9% of all sequences, respectively (Fig. 4a). Phylogenetic analysis of Tulasnellaceae OTUs revealed that both TU1 and TU2 formed well supported monophyletic clades with previously identified orchid mycobionts of epiphytic orchids widely distributed across the Americas (TU1) and Asia (TU2), suggesting that *T. marianense* can associate with cosmopolitan fungi with wide geographic distributions.

Although Tulasnellaceae are broadly distributed and commonly reported in OMF associations globally (Jacquemyn et al. 2017; Oberwinkler et al. 2017), their presence in Guam is confirmed here for the first time, along with their functional role as OMF symbionts. Among the few reports available on OMF from isolated islands, Martos et al. (2012) analyzed 77 different orchid species on Réunion Island, a volcanic island in the Indian Ocean, off the southeast coast of Africa. In their study, Tulasnellaceae were the primary fungal associates in 34 epiphytic orchid species, followed by Serendipitaceae. The authors proposed that although some orchids may maintain ancient associations with specific fungi, these relationships can become more flexible over time, particularly when there is no strong phylosymbiotic signal linking specific orchids to specific fungal associates. Similarly, Rammitsu et al. (2021a) reported a highly specific mycorrhizal association in *Dendrobium okinawense* from Okinawa, a subtropical island in southern Japan. In their study, *D. okinawense* consistently associated with a single Tulasnellaceae OTU across multiple populations. This level of specificity contrasts with other *Dendrobium* species, which have been observed to associate with a broader range of fungal taxa.

Through our symbiotic germination trials of the two Tulasnellaceae strains, TU1 and TU2, we confirmed their mycorrhizal capacity in facilitating seed germination and advanced protocorm development in *T. marianense*. Though slightly more abundant, TU1 was found to have a smaller effect in promoting seed germination and protocorm development compared to TU2 which facilitated nearly all seeds to the advanced protocorm stage (Stage 4) within 33 days (Fig. 8). Regardless, both strains of Tulasnellaceae had clear positive effects relative to the control treatment where nearly all seeds remained ungerminated.

Differences in mycorrhizal capacity among fungal strains isolated from the same orchid species have been reported in other studies and are often linked to orchid ontogeny, where distinct OMF may support different stages of orchid development (Fernández et al. 2023; McCormick et al. 2004; Meng et al. 2019). In our mixed sample of spontaneous seedlings and mature *T. marianense*, both TU1 and TU2 were identified at similar frequencies across growth stages. However, our in vitro germination assays suggest TU1 may be less effective at supporting late-stage seedling development (Fig. 8). This discrepancy between field observations and laboratory results could indicate that TU1 forms mycorrhizal associations less readily under our culture conditions, supports seedling growth more slowly than TU2, or requires specific environmental factors not replicated in our experimental setup. Therefore, while both OTUs appear to associate with *T. marianense* across its natural life history, their functional roles and efficiency likely shift with environmental context. Moreover, while some host tree species were observed to harbor both OTUs, others supported only one, indicating that TU1 and TU2 may be undergoing niche partitioning driven by microhabitat differences among host trees (Li et al. 2021; Rasmussen et al. 2015). Together, the complementary niches of TU1 and TU2 may help explain the broad distribution of *T. marianense* across many host trees and habitats in Guam.

While our study reveals that the leafless epiphytic orchid *T. marianense* in Guam is highly specific to Tulasnellaceae fungi, these findings contradict several studies indicating a highly specialized association of the leafless epiphytic orchid group with Ceratobasidiaceae fungi, including species such as *Campylocentrum fasciola*, *Ca. filiforme* (Otero et al. 2002), D. *lindenii* (Hoang et al. 2017; Mújica et al. 2018), *T. glandulosum* (Rammitsu et al. 2019; Yagame and Yamato 2009) and *T. obtusum* found across Asia and the Americas (Irawati 2009). Our findings also conflict with recent high-throughput sequencing studies of OMF communities in other leafless epiphytic orchids in China, which identified Ceratobasidiaceae as the dominant mycorrhizal associates in *T. glandulosum*, *Phalaenopsis wilsonii*, *P. braceana*, *Chiloschista exuperei*, *C. guangdongensis*, *C. yunnanensis*, and *C.* aff. *viridiflava* (Qin et al. 2021). Additionally, symbiotic germination trials in these studies demonstrated that Ceratobasidiaceae strains more effectively supported seed germination and seedling development in these leafless epiphytes, whereas Tulasnellaceae strains failed to promote germination (Hoang et al. 2017; Irawati 2009; Qin et al. 2021).

In our study, Ceratobasidiaceae fungi accounted for only 13.1% of sequences from *T. marianense* (Fig. 4a). Phylogenetic analysis revealed that the most common Ceratobasidiaceae OTUs (CE1 and CE5) clustered with fungi from the leafless *T. glandulosum* from Japan (Rammitsu et al. 2019; Yagame and Yamato 2009), while CE4 was closely related to *Ceratobasidium* from the critically endangered, leafless epiphyte *Dendrophylax lindenii* from Florida and Cuba (Unruh et al. 2019). While these observations demonstrate that *T. marianense* is capable of associating with Ceratobasidiaceae related to those identified in other leafless epiphytes, CE1 comprised only 4.5% of sequences, CE4 represented 0.5%, and CE5 was detected just once (0.02%), collectively suggesting a minor role in symbioses. In other leafless epiphytes, Ceratobasidiaceae typically dominate mycorrhizal associations (Hoang et al. 2017; Irawati 2009; Johnson et al. 2023; Mújica et al. 2018; Otero et al. 2002; Qin et al. 2021; Rammitsu et al. 2019; Yagame and Yamato 2009).

Considering their obligate dependence on mycorrhizal associates, orchids colonizing remote islands must either co-disperse with their fungal associates or establish associations with compatible fungi already present in the environment. Yet according to classical island biogeography theory (Carlquist 1974; MacArthur and Wilson 2001), remote oceanic islands such as Guam are expected to harbor reduced species pools and should therefore favor species with generalist strategies, particularly those with obligate symbioses such as orchids and their mycorrhizal fungi. In these island systems with limited fungal diversity, mycorrhizal associations are predicted to favor a few cosmopolitan and readily available fungal associates.

The highly specific mycorrhizal association of *T. marianense* in Guam with Tulasnellaceae fungi, a globally widespread fungal group, rather than the expected Ceratobasidiaceae fungi suggests that this orchid may have adapted to associate with the most accessible and abundant fungal symbiont upon colonizing the island. Environmental conditions in Guam, characteristics of host trees, or physiological traits of *T. marianense* may have further favored these Tulasnellaceae associations. Studies in other continental island systems have shown similar patterns in the persistence of mycorrhizal specificity through cosmopolitan and locally accessible Tulasnellaceae or other fungal taxa (Martos et al. 2012; Otero et al. 2002, 2007b; Rammitsu et al. 2021a; Swift et al. 2019). Alternatively, the minor associations with Ceratobasidiaceae fungi in *T. marianense* may reflect later introductions of these fungi to the island after the orchid had already established its primary symbiotic relationship with Tulasnellaceae, possibly explaining their limited detection in this study.

Notably, although Guam’s prevailing trade winds originate from the east and northeast, they can shift to southwesterly winds during the monsoon season (June–December) (Lander 1996; Lander and Guard 2003). This seasonal wind reversal provides a possible dispersal route from New Guinea, the center of distribution for *Taeniophyllum* (Schlechter 1982; Seidenfaden and Wood 1992), suggesting that wind-driven seed dispersal could have facilitated the orchid’s arrival to Guam. Whether Tulasnellaceae fungi arrived via the same pathway or were already established through earlier colonization events remains unclear. Further investigation of mycorrhizal associations in other Pacific island *Taeniophyllum* endemics could reveal whether Tulasnellaceae specificity is conserved across islands or reflects a stepping-stone evolutionary pattern. Regardless of the underlying mechanism, this adaptive flexibility in mycorrhizal associations may help explain the broader distribution and persistence of *T. marianense* across diverse habitats in Guam.

Our study suggests that mycorrhizal specificity may not be a limiting factor for orchid establishment on remote oceanic islands. Rather than adopting overt generalist strategies, *T. marianense* may achieve successful establishment by associating with widely distributed and locally available fungal symbionts present in Guam. This adaptive flexibility may serve as a critical strategy for orchid survival and persistence in resource-limited island ecosystems, underscoring the importance of symbiotic adaptability in facilitating successful establishment on remote oceanic islands.

## Supplementary Information

Below is the link to the electronic supplementary material.

Supplementary Material 1

## Data Availability

Sequences generated from this study are available on the NCBI GenBank database for Tulasnellaceae (PP938874–PP938878), Ceratobasidiaceae (PP938889–PP938894) and Serendipitaceae (PP916010–PP916013) OTUs. The sequence alignments and phylogenetic trees are available on the Dryad data repository: 10.5061/dryad.kprr4xhd3.
