# Supplementary material for: Mycorrhizal specialization for Tulasnellaceae fungi in Taeniophyllum marianense, a leafless epiphytic orchid native to Guam
Source: J Plant Res. 2026 Mar 9;139(3):409–24. doi: 10.1007/s10265-026-01699-z (PMC13197293; doi:10.1007/s10265-026-01699-z)
Supplement: Supplementary file 1 — Supplementary Material 1 [file 10265_2026_1699_MOESM1_ESM.pdf]

Supplementary information

Mycorrhizal specialization for Tulasnellaceae fungi in *Taeniophyllum marianense*, a leafless epiphytic orchid native to Guam

Journal of Plant Research

Michael Angelo Paragas Fernandez[1,4], Yuki Ogura-Tsujita[2,3], & Mari Marutani[1]

[1] College of Natural and Applied Sciences, University of Guam, Mangilao, Guam, 96923, USA

[2] Faculty of Agriculture, Saga University, 1 Honjyo-machi, Saga 840-8502, Japan

[3] United Graduate School of Agricultural Sciences, Kagoshima University, 1-21-24 Korimoto, Kagoshima 890-8580, Japan

[4] Current address: Pacific Biosciences Research Center, Hawai'i at Mānoa, Honolulu, HI 96822, USA

Corresponding author: Yuki Ogura-Tsujita

ytsujita@cc.saga-u.ac.jp

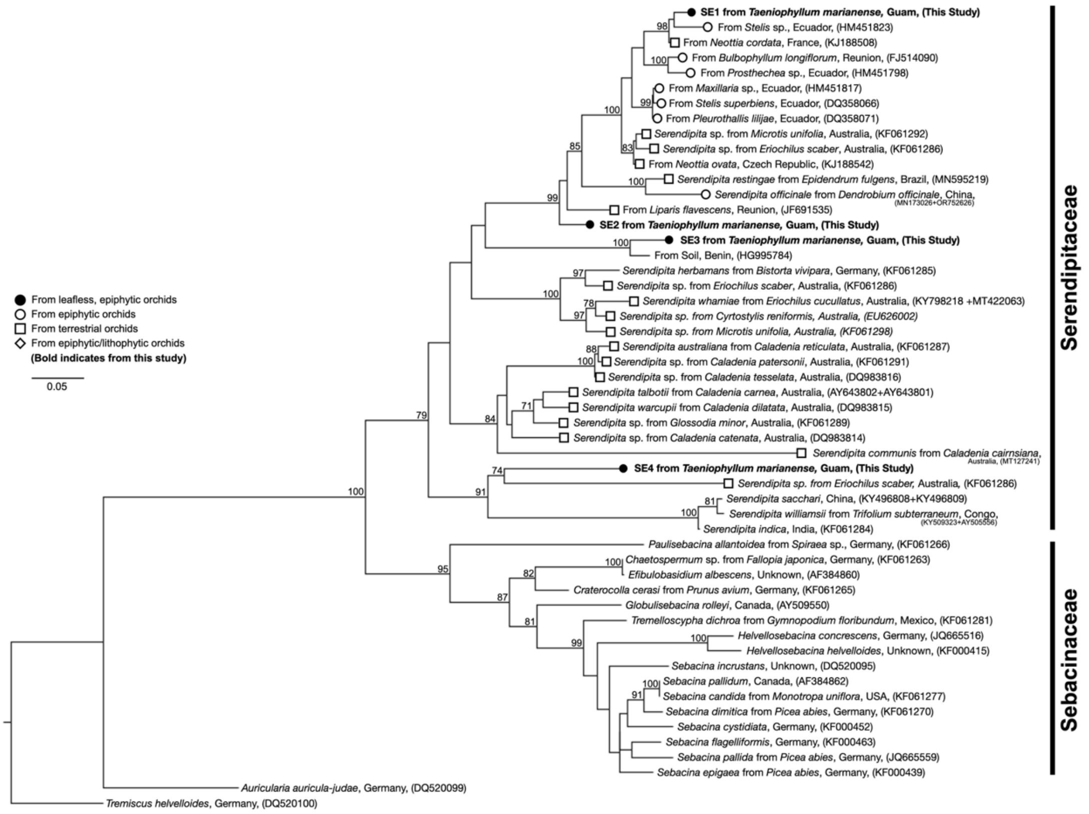

Fig. S1 Maximum likelihood tree for Serendipitaceae OTUs identified in *Taeniophyllum marianense* using partial ITS2 and complete 28s regions. Symbols indicate type of orchid host. No symbol indicates an unknown or non-orchid origin. Only bootstrap values greater than 70% are shown. *Tremiscus helvelloides* and *Auricularia auricula-judae* were selected as the outgroup taxa. The tree is drawn to scale and branch length is proportional to the number of substitutions per site. The final dataset comprised 537 bp.

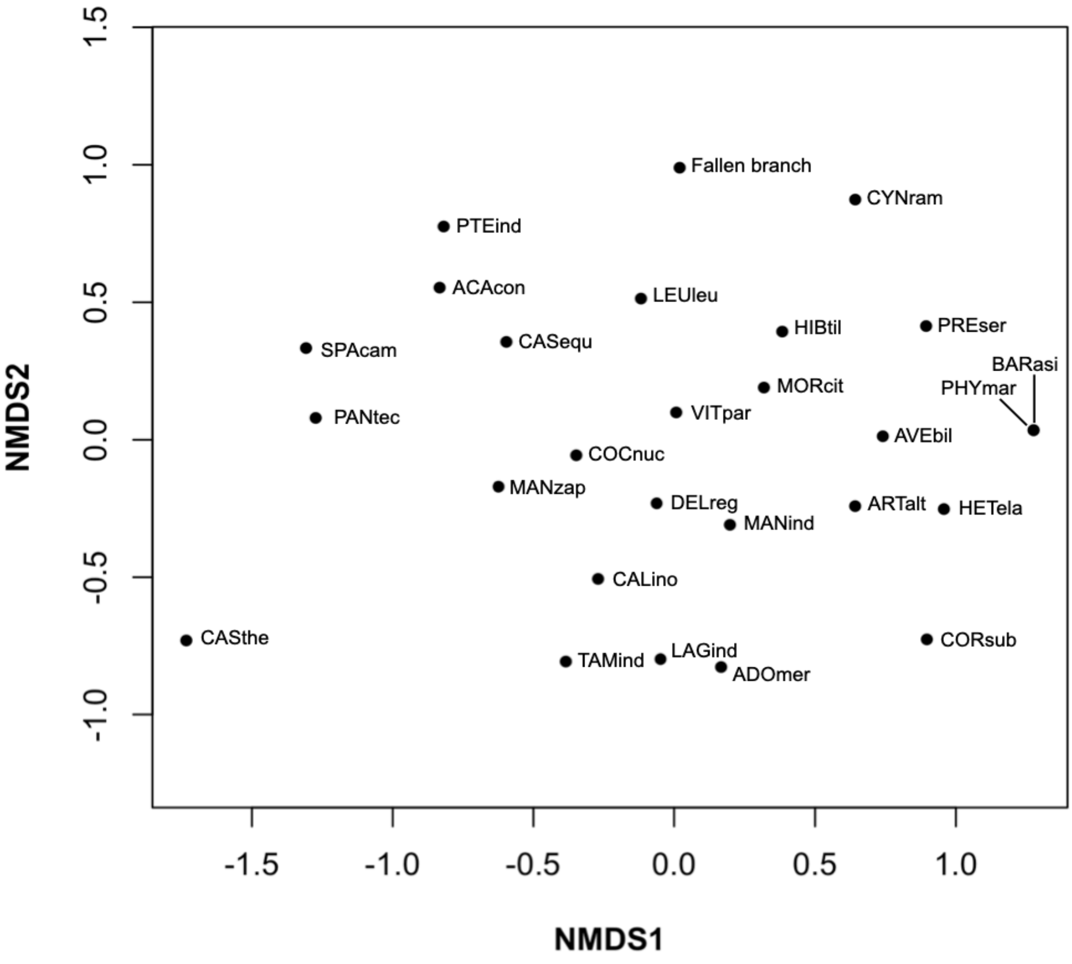

Fig. S2 Non-metric multidimensional scaling (NMDS) plot of mycorrhizal communities of *Taeniophyllum marianense*. The effect of host tree species on mycorrhizal communities (stress = 0.142). ACAcon: *Acacia confusa*; ADOmer: *Adonidia merrillii*; ARTalt: *Artocarpus altilis*; BARasi: *Barringtonia asiatica*; AVEbil: *Averrhoa bilimbi*; CALino: *Calophyllum inophyllum*; CASequ: *Casuarina equisetifolia*; COCnuc: *Cocos nucifera*; CORsub: *Cordia subcordata*; CYNram: *Cynometra ramiflora*; DELreg: *Delonix regia*; HETela: *Heterospathe elata*; HIBtil: *Hibiscus tiliaceus*; LAGind: *Lagerstroema indica*; LEUleu: *Leucaena leucocephala*; MANind: *Mangifera indica*; MANzap: *Manilkara zapota*; MORcit: *Morinda citrifolia*; PANtec: *Pandanus tectorius*; PHYmar: *Phyllanthus mariannensis*; PREser: *Premna serratifolia*; PTEind: *Pterocarpus indicus*; SPAcam: *Spathodea campanulata*; TAMind: *Tamarindus indica*; CASthe: *Cascabela thevetia*; VITpar: *Vitex parviflora,* Fallen branch.

Table S1. List of other Basidiomycota OTUs detected from *Taeniophyllum marianense* root samples, including OTU ID, number of sequences, best BLAST match name and accession number, query coverage (%), and percent identity (%). Fungal ITS amplicon sequences from root samples were clustered into OTUs based on 97% sequence similarity.

OTU ID	No. of sequences	Best BLAST match name	Best BLAST accession no.	Query coverage (%)	Percent identity (%)
BA1	15	Uncultured Septobasidiaceae	MH0055887	81.8	97.8
BA2	8	Uncultured Basidiomycota	U65610	86.2	93.8
BA3	7	Uncultured Septobasidiaceae	HM209415	39.9	91.0
BA4	5	Uncultured Septobasidiaceae	MH005911	82.6	87.8
BA5	3	*Auricularia* sp.	ON715753	99.3	92.4
BA6	3	*Efibula tuberculata*	MZ636969	99.9	81.8
BA7	2	Uncultured Basidiomycota	GU328610	100	86.4
BA8	2	*Gymnopilus* sp.	MZ997039	100	87.4
BA9	2	*Pallidohirschioporus versicolor*	OQ504334	36.4	86.7
BA10	2	*Trechisporales* sp.	LC327027	98.8	99.5
BA11	2	Uncultured Septobasidiaceae	MH005909	83.2	84.5
BA12	1	Uncultured *Alnicola*	JQ890290	72.8	83.8
BA13	1	Uncultured Schizophyllaceae	LS987628	97.1	84.4
BA14	1	Uncultured Trechisporales	JF691365	99.3	84.4
BA15	1	*Clitopilus baronii*	PQ652399	100	95.7
BA16	1	*Phlebiella* sp.	KP783450	99.9	81.6
BA17	1	*Marasmiellus* sp.	MW220847	87.4	99.4
BA18	1	*Marasmius leveilleanus*	MF189067	100	96.1
BA19	1	*Dendrothele bispora*	OM238172	100	96.9
